# A study on the timing of uterine artery embolization followed by pregnancy excision for cesarean scar pregnancy: a prospective study in China

**DOI:** 10.1186/s12884-021-04180-y

**Published:** 2021-10-15

**Authors:** Liping Yu, Bikang Yang, Qinyang Xu, Yincheng Teng, Zhuowei Xue

**Affiliations:** The Department of Obstetrics and Gynecology, Shanghai Jiao Tong University Affiliated Sixth People’s Hospital, 600 Yishan Road, Shanghai, 200233 China

**Keywords:** Cesarean scar pregnancy, Uterine artery embolization, Dilation and curettage, Time interval

## Abstract

**Background:**

Cesarean scar pregnancy (CSP) remains a sporadic and special form of ectopic pregnancy in which the fertilized ovum is implanted on a previous cesarean scar within 12 weeks. This study aims to evaluate the optimal time interval between uterine artery embolization (UAE) and curettage modalities in order to provide the best clinical outcomes.

**Methods:**

From January 2018 to December 2020, we recruited 61 patients with CSP. They were randomly divided into two groups depending on whether the time interval between UAE and dilatation and curettage (D&C) requires additional hospitalization: 31 patients received prophylactic UAE followed by D&C on the same day (0–12 h; group A) and 30 patients need hospitalization (12–72 h; group B). The clinical characteristics, diagnostic data, and outcomes of the two groups were compared and analyzed.

**Results:**

A total of 59 (96.72%) cases had responded well to the first treatment. One patient in each arm undergone retreatment, but none of the 61 patients needed additional hysterectomy. There was no considerable relationship between the two groups with respect to the intraoperative hemorrhage during D&C, serum index (containing β-hCG, hemoglobin, CRP, and D-dimer) on the first day after D&C, side effects (containing fever and abdominal pain), renal, hepatic, and coagulation function, time of CSP residual mass disappearance, and hospitalization cost. The time of serum β-hCG resolution after surgery was 41.22 ± 14.97 days in group A and 66.67 ± 36.64 days in group B (*P* = 0.027), and group A treatment resulted in a shorten hospital stay as compared with group B (4.81 ± 2.74 days vs. 6.80 ± 2.14 days, *P* <  0.001). However, the average hourly serum β-hCG decrease rate within 24 h and the leukocytes on the first day after D&C in group B were superior than in group A (*P* <  0.050).

**Conclusion:**

For patients with CSP, UAE followed by D&C on the same day (0–12 h) appears to have more advantages in hospitalization and recovery time, while the long time interval (12–72 h) may have a lower risk of inflammation and a more rapid decrease in serum β-hCG level within 24 h after D&C surgery. The treatment of CSP should be individualized based on the conditions of patients.

## Background

Most pregnant women in China need to undergo delivery in hospital, around 20% do so by cesarean section (CS). CS is associated with an increased trend of uterine scar niche [1, 2]. There has been an increasing prevalence in the occurrence of cesarean-related iatrogenic complications, especially cesarean scar pregnancy (CSP), which leads to a diagnostic and therapeutic challenge for obstetricians [3, 4]. CSP is believed to occur when the fertilized ovum implanted on fibrous incision site of myometrium in a prior CS within 12 weeks [5]. Currently, novel treatments of CSP were developed, varying from medical management and minimally invasive surgical approach [6–9].

A wide range of treatment methods to terminate a CSP is available. Treatment management is usually selected by the obstetrician’s experience, availability of treatment means, and facilities, especially in China [10]. However, there are still no widely agreed upon or adopted treatment procedures [10]. With limited therapeutic effectiveness of CSP, D&C and UAE alone should not be first-line treatments [11]. UAE combined with D&C has a high success rate [12]. The previous studies have reported that the time interval between the UAE and D&C is set for 24 to 72 h [13–15]. Recent research has added support to an updated idea: that a time interval not be delayed longer than 72 h [16]. Therefore, the optimal time interval is of particular importance.

We initiated a prospective clinical study comparing the characteristics of CSP patients who were performed prophylactic UAE within either 12 h or 12–72 h followed by D&C regimen. The current study aimed to evaluate the optimal time interval between UAE and D&C regimen, that is, whether the procedure is completed on the same day or additional hospitalization, and to identify the most effective treatment. The findings from our results might provide appropriate treatment for patients with this iatrogenic disease.

## Methods

### Subjects criteria

This study was conducted among patients with CSP in the Department of Obstetrics and Gynecology, Shanghai Sixth People’s Hospital Affiliated to Shanghai Jiaotong University, Shanghai, China, between January 2018 to December 2020. The protocol was approved by the Institutional Review Board of Shanghai Sixth People’s Hospital Affiliated to Shanghai Jiaotong University, and written informed consent was obtained from all participants before enrollment. The inclusion criteria were satisfied: (1) a history of cesarean delivery and amenorrhea; (2) ultrasonography or magnetic resonance imaging (MRI) confirmed CSP; and (3) prophylactic treatment with UAE is accepted. Exclusion criteria included: (1) severe cardiopulmonary comorbidity; (2) hematological disease; (3) hepatic and renal dysfunction; (4) unstable vital signs; (5) inevitable or incomplete abortion. All participants with CSP were randomly allocated into two groups to receive prophylactic UAE within 12 h (A group, *n* = 31) or 12–72 h (B group, *n* = 30) followed by D&C.

### Management

All patients underwent preventive UAE. In details, UAE was performed under local anesthesia by two experienced interventional specialists. The patient was placed in a supine position. The catheterization was completed through a percutaneous right femoral artery puncture using the Seldinger technique. 5-F catheter introducer (5-F, TERUMO, Japan) was inserted into the bilateral iliac arteries for arteriography, respectively. The frontal radiograph showed that the bilateral uterine arteries were obviously augmentation and circuitous, the uterine spiral arteries were increased, and there was no arteriovenous shunt. After finding the opening of the uterine artery, the micro-catheters (135/150 cm, Asahi, Japan) were advanced into the distal end of the uterine artery. Embolization was carried out by injection of gelatin sponge particles (300–500 μm) (S410GH, BioSpere Medical, USA) into both uterine arteries. Digital subtraction arteriography was conducted to confirm that bilateral uterine arteries were completely embolized.

All patients with CSP were performed D&C after UAE within 12 h or 12–72 h to remove the conception and blood clots. (1) During D&C guided by ultrasonography, the gestational sac and clot tissue were removed via oval forceps or vacuum curette. The suction head entered the uterine cavity along the bottom cervical canal, sucked the decidua of bilateral uterine cornua on the posterior wall of the uterus, and finally sucked the anterior uterine wall. (2) The incision site was operated gently, and the gestational sac tissue was cleaned under the guidance of ultrasonography. (3) The myometrium mass in incision waited to absorb or fall off. (4) Blood loss during the operation was measured by a measuring cup.

The serum beta human chorionic gonadotropin (β-hCG) level and routine blood test, liver and renal function tests were measured the day before and after surgery. The size of the gestational sac mass in the incision site was measured by transvaginal ultrasound.

### Investigation content

The clinical characteristics of 61 patients with CSP were investigated. The investigation content included the maternal age, the body mass index (BMI), the gestational age, the number of prior pregnancy (containing gravidity, abortion, CS, living, premature), the interval between CS and CSP, the gestational sac diameter, the fetal cardiac activity, embryo length, CSP type, the estimated intraoperative hemorrhage, the serum β-hCG levels before and after surgery, routine blood level before and after surgery, the side effects, the hospitalization time, and the treatment cost. There are three types of CSP confirmed by ultrasonography or MRI: type I with progression to intrauterine direction (endogenic type); type II with progression toward the bladder (exogenic type); and type III formed by uterine abortion or incomplete medical abortion (mixed type) [5, 17].

All patients were followed up for up to 6 months after surgeries. They were followed up to review their serum β-hCG levels and clinical status until the normalization of serum β-hCG (< 5.00 IU/L). Average hourly serum β-hCG decrease rate within 24 h after D&C was defined as UAE-preoperative and D&C-postoperative serum β-hCG decline rate divided by D&C-postoperative detection time (within 24 h). Transvaginal ultrasound was performed at 1 week, 2 weeks, and menstruation to review the residual mass in the incision site until disappearance (the hydrops or mass diameter < 1.00 cm). Days for β-hCG to normal and the time for CSP mass disappearance were recorded. Information about their any other symptoms was also observed. Variables included average hourly serum β-hCG decrease rate within 24 h after D&C, the time of CSP residual mass disappearance, and the time of serum β-hCG resolution.

### Statistical analysis

All of the data analyses were conducted using SPSS 23.0 software (SPSS, Inc., USA). The differences in numerical data among these two groups were presented in mean ± standard deviation (SD) and compared using the student’s t-test. If the assumptions of normality and homogeneity of numerical data are not satisfied, the Mann–Whitney U test was used. The categorical data were analyzed via the χ^2^ test or the Fisher’s exact test. A value of *P* < 0.050 was considered statistically significant for paired comparisons.

## Results

### Clinical characteristics

In the 3 years, a total of 61 patients with CSP was enrolled in our study. Preoperative laboratory test results of all patients were normal and eligible. There were no significant differences in the baseline parameters of subjects in the two groups (*P* > .050) (Table 1). The mean maternal age was 33.65 years in group A and 32.57 years in group B. Their BMI ranged between 15.60 and 44.10 kg/m^2^, with a mean of 21.64 kg/m^2^ in group A and 22.34 kg/m^2^ in group B. The mean serum β-hCG, leukocytes, hemoglobin, D-dimer, and C-reactive protein (CRP) before UAE was were similar (*P* > .050). The median gravidity, term birth, preterm birth, abortion, live birth, and CS in group A and B were 3.00 (range 1.00–11.00) vs. 3.00 (range 1.00–7.00), 1.00 (range 0–2.00) vs. 1.00 (range 0–2.00), 0 (range 0–1.00) vs. 0 (range 0–1.00), 2.00 (range 0–9.00) vs. 2.00 (range 0–6.00), 1.00 (range 1.00–2.00) vs. 1.00 (range 1.00–2.00), and 1.00 (range 1.00–2.00) vs. 1.00 (range 1.00–2.00), respectively. Other clinical characteristics and previous history of the patients were no significant differences.

**Table 1:** Patient characteristics and demographics

Characteristic	Group A (*n* = 31)	Group B (*n* = 30)	*P*^a^ value
Maternal age (years)	33.65 ± 5.79	32.57 ± 5.67	0.465
BMI (kg/m^2^)	21.64 ± 3.04	22.34 ± 4.72	0.488
Amenorrhea (days)	45.90 ± 9.35	49.63 ± 12.08	0.182
Serum β-hCG before UAE (× 10^4^ IU/L)	5.46 ± 6.04	4.71 ± 4.56	0.599
Leukocytes before UAE (× 10^9^/L)	7.61 ± 2.07	7.28 ± 1.74	0.502
Hemoglobin before UAE (g/L)^d^	119.40 ± 12.28	122.89 ± 10.97	0.260
D-dimer before UAE (mg/L)^d^	0.39 ± 0.45	0.67 ± 1.36	0.344
CRP before UAE (mg/L)^d^	2.88 ± 2.10	2.59 ± 1.35	0.748
Preoperative gestational sac diameter (cm)	2.65 ± 0.95	2.48 ± 1.15	0.553
Preoperative ultrasound mass (cm)	2.68 ± 0.87	3.07 ± 0.96	0.103
Preoperative fetal pole (mm)	3.11 ± 3.76	3.91 ± 5.89	0.534
Preoperative fetal cardiac activity [n (%)]	14.00 (45.16)	12.00 (40.00)	0.684^c^
Gravidity [times, median (range)]	3.00 (1.00–11.00)	3.00 (1.00–7.00)	0.380^b^
Term birth [times, median (range)]	1.00 (0–2.00)	1.00 (0–2.00)	0.475^b^
Preterm birth [times, median (range)]	0 (0–1.00)	0 (0–1.00)	0.329^b^
Abortion [times, median (range)]	2.00 (0–9.00)	2.00 (0–6.00)	0.526^b^
Live birth [times, median (range)]	1.00 (1.00–2.00)	1.00 (1.00–2.00)	0.307^b^
CS [times, median (range)]	1.00 (1.00–2.00)	1.00 (1.00–2.00)	0.393^b^
Interval between CS and CSP (years)	7.03 ± 5.15	6.00 ± 4.45	0.469^b^
CSP type [n (type I/II/III)]	13/15/3	16/11/3	0.616^c^

*BMI* body mass index, *CRP* C-reactive protein, *CS* cesarean section, *CSP* cesarean scar pregnancy, *UAE* uterine artery embolization, *β-hCG* beta human chorionic gonadotropin

*Indicates that the item was significantly different between the two groups

^a^Analyzed by the *t* test; ^b^Mann-Whitney U test; and ^c^χ^2^ test or Fisher’s exact test. ^d^Missing data for some of the patients

### Clinical outcomes and follow-up

Clinical outcomes were presented in Table 2. There was no statistically significant difference in the success rate in the initial treatment between the two groups (96.77% vs. 96.67%, *P* = 1.000). One patient in each group readmitted to undergo a treatment. None required a hysterectomy. Of these 2 patients, one patient with CSP type II in group A was a 29-year-old woman (term birth 1; abortion 3; CS 1) and admitted to our hospital with the complaint of amenorrhea for 39 days. The ultrasonography revealed an obvious gestational sac (diameter > 3.00 cm) without fetal cardiac activity. She was performed prophylactic UAE 3 days after admission and followed by D&C 4 h later. Blood routine reexamination showed an upward trend in serum β-hCG after discharge (12,939.00 IU/L in 2 days; 15,807.00 IU/L in 9 days; 22,142.00 IU/L in 11 days), and ultrasonography indicated that the scar pregnancy changed from type II to type III. Fourteen days later, she readmitted to receive treatment with laparoscope and metroplasty. Another case (term birth 1; abortion 1; CS 1) in group B showed a downward trend in reexamination of serum β-hCG after discharge. There was still a large range of mixed echoes at the uterine incision and the lower uterine segment, and persistent vaginal bleeding for 1 month. A second D&C was performed in this patient owing to CSP residual mass (diameter > 3.00 cm).

**Table 2:** Outcomes of two groups

Characteristic	Group A (*n* = 31)	Group B (*n* = 30)	*P*^a^ value
Intraoperative hemorrhage during D&C (mL)	40.35 ± 45.89	55.40 ± 118.28	0.512
Serum β-hCG on the first day after D&C (× 10^4^ IU/L)^d^	2.11 ± 2.26	1.01 ± 0.99	0.078
Leukocytes on the first day after D&C (×10^9^/L)^d^	13.19 ± 4.30	9.83 ± 2.65	0.006^*^
D-dimer on the first day after D&C (mg/L)^d^	1.29 ± 1.07	1.33 ± 1.21	0.932
Hemoglobin on the first day after D&C (g/L)^d^	114.14 ± 11.50	110.61 ± 9.11	0.301
CRP on the first day after D&C (mg/L)^d^	50.20 ± 32.30	59.91 ± 41.40	0.469
Success rate in the first treatment [n (%)]	30 (96.77)	29 (96.67)	1.000^c^
Hysterectomy [n (%)]	0 (0)	0 (0)	1.000^c^
Fever [n (%)]	28 (90.32)	24 (80.00)	0.301^c^
Abdominal pain [n (%)]	31 (100.00)	30 (100.00)	1.000^c^
Anomalous renal function [n (%)]^d^	4 (50.00)	0 (0)	0.467^c^
Anomalous hepatic function [n (%)]^d^	0 (0)	0 (0)	1.000^c^
Anomalous coagulation function [n (%)]^d^	1 (6.25)	0 (0)	1.000^c^
Average hourly serum β-hCG decrease rate within 24 h after D&C (%/h)^d^	3.82 ± 0.73	10.97 ± 17.42	0.028^b *^
Time of CSP residual mass disappearance (days)^d^	20.32 ± 14.36	19.00 ± 12.56	0.896^b^
Time of serum β-hCG resolution (days)^d^	41.22 ± 14.97	66.67 ± 36.64	0.027^b *^
Hospitalization cost (×10^4^ CNY)	1.97 ± 0.51	1.78 ± 0.51	0.170^b^
Hospitalization time (days)	4.81 ± 2.74	6.80 ± 2.14	< 0.001^b *^

*CNY* China yuan, *CRP* C-reactive protein, *CSP* cesarean scar pregnancy, *D&C* dilatation and curettage, *β-hCG* beta human chorionic gonadotropin

*Indicates that the item was significantly different between the two groups

^a^Analyzed by *t* test; ^b^Mann-Whitney U test; and ^c^χ^2^ test or Fisher’s exact test. ^d^Missing data for some patients

There was no significant differences between the two groups with respect to the intraoperative hemorrhage during D&C, serum β-hCG, hemoglobin, CRP, and D-dimer on the first day after D&C, time of CSP residual mass disappearance, and hospitalization cost. As unexpected, the time of serum β-hCG resolution after surgery was 41.22 ± 14.97 days in group A and 66.67 ± 36.64 days in group B (*P* = 0.027), and the hospitalization time was 4.81 ± 2.74 in group A and 6.80 ± 2.14 in group B (*P* < 0.001). However, the average hourly serum β-hCG decrease rate within 24 h and the leukocytes on the first day after D&C in group B were significantly superior than in group A (*P* < 0.050).

We examined the incidence of complications among the two groups. Fever and abdominal pain were common. According to the results, 28 patients in group A and 24 patients in group B had fever (*P* = 0.301), and all patients reported abdominal pain. Only one patient in group A underwent anomalous coagulation function. She was a 31-year-old woman (gravidity 3; CS 1) with 51 days of amenorrhea and admitted to our hospital. She was offered group A procedure in which prophylactic UAE within 4 h followed by D&C, intraoperative hemorrhage during D&C only had 10.00 mL. In group A, 4 patients shown anomalous renal function (compared with group B, *P* = 0.467). No patients in both groups had an anomalous hepatic function (*P* = 1.000).

## Discussion

In the present study, we showed that CSP patients treated with UAE followed by D&C on the same day (0–12 h; group A) or hospitalization (12–72 h; group B) were both results in good response. Both cohorts had less complications and intraoperative hemorrhage, indicating that UAE followed by D&C is a safe and effective regimen for treatment CSP patients. To our knowledge, this is the first prospective study for the comparative analysis of the time interval between the UAE and D&C in recent years.

For most patients with CSP, if they insist on intrauterine pregnancy, they can finally bring the fetus to term and complete the delivery. However, the treatment of CSP is the preferred intervention, which should be carried out termination of CSP immediately after diagnosis. Advanced diagnostic techniques and prompt managements are crucial to avoid obstetric complications [11, 18]. Therefore, in our study, we provided immediate diagnosis and treatment for all patients with CSP. Approximately 40% of clinical manifestations of CSP are incidental detection or asymptomatic, others range from vaginal bleeding, abdominal pain, and hemodynamic instability. Acute abdominal pain with vaginal bleeding should raise concern for impending rupture [19]. The first-line and second-line treatments are still not good enough. Medication treatment is less invasive, and surgical procedures have been adopted in the treatment of CSP. Surgical procedures include laparoscope, D&C, hysteroscopy, or in case of a patient with massive hemorrhage, hysterectomy has been performed [12]. Hysterectomy has been considered to be the emergency surgery as removal of CSP can directly avoid the residual trophoblasts and, thus, recurrence of CSP. Even so, the uterine conserving treatment is preferred [20]. Until now, many methods have been established. Clinically, UAE combined with D&C has been commonly used for CSP treatment in China. This method provided effective results as shown in our study.

Considering the urgency of some patients with CSP for the operation, we prospectively designed a comparison between D&C after UAE on the same day (0–12 h) and an extra hospitalization stay for 1–3 days after conventional UAE. Although in most clinical studies, the time interval between UAE and D&C is the latter [3, 21, 22]. Generally, the time interval is within 12 h, that is, the UAE treatment can be performed in the morning, and D&C can be completed in the afternoon of the same day. Patients do not need to be hospitalized for the waiting time after UAE, which means that they can be discharged the next day after completing the CSP treatment on the same day. The embolization time of UAE is more than 12 h (12–72 h), and the patients must be hospitalized to wait for the next D&C. Patients may have to pay extra for medical expenses and hospitalization stay.

Our data shown that UAE within 12 h or 12–72 h followed by D&C was successful in terminating CSP and retaining the patient’s uterus and future fertility. This is consistent with the conclusion of Wang et al. [16]. Management of UAE followed by D&C might be a priority option because of the low intraoperative hemorrhage. Similar results have been reported [13, 23]. The same between our results and those of Qian et al. might be owing to the patients were performed prophylactic UAE followed by D&C to prevent massive hemorrhage in research. A larger CSP mass diameter (≥ 6.00 cm), a greater gestational age (≥ 56 days), parity, fetal cardiac activity, and embolic agent diameter were risk factors for massive hemorrhage (≥ 500 mL) during UAE followed by D&C [14, 15]. In this study, the case of the maximum intraoperative hemorrhage during D&C is only 450 mL. This patient has gestational sac diameter of less than 3 cm, a shorter gestational age (50 days), and no fetal cardiac activity. Our results might not applicable to patients with above risk factors.

Most CSP studies are mainly limited to pathology reports or small case series described in the literature, with no recognized guidelines or standard managements on the preferred program of CSP therapy since various methods are available [24, 25]. In the UAE combined with D&C, the timing of surgery after intervention is still controversial. In this study, the shorter time intervals (within 12 h) between UAE and D&C display clinical advantages, which may offer faster recovery times and shorter hospital stay. Transitory obstruction of bilateral uterine artery may induce stress inflammatory response, resulting in a significant increase in the number of leukocytes. The long interval group (UAE within 12–72 h followed by D&C) shown that the rate of decline of serum β-hCG is more obvious within postoperative 24 h, and the number of leukocytes on the first day after D&C was lower. There was no significant difference in intraoperative hemorrhage in both groups. The advantages of long blocking time after UAE is that it has enough time to obstruct the bilateral uterine arteries and temporarily weaken the blood supply of the placenta, which can assist in embryonic necrosis. Frail embryos can be removed more effectively after D&C, thereby reducing the intraoperative and postoperative risks as well as improving the success rate of surgery [23, 26].

CSP patients of reproductive age are more concerned with subsequent fertility after UAE. Several reports suggested that UAE might be associated with adverse effects on fertility, including increased rate of miscarriage, preterm delivery, intra-uterine growth restriction, and post-partum hemorrhage. Some obstetricians did not recommend UAE for these patients [27]. However, a study showed that 43.8% (7/16) of CSP patients conceived after UAE + D&C treatment without any complications and had a 100.0% (7/7) live birth rates [23]. Qiu Jian et al. reported that 23 CSP patients desired to conceive in future after treatment with UAE and D&C, and 87.9% (20/23) patients resulted in subsequent conceptions. Only 25.0% (5/20) patients underwent uneventful parturition without any complications. Other patients underwent pregnancy with placenta previa/accreta, miscarriage, recurrent CSP, and infertility [28]. In a case study, a patient diagnosed as CSP underwent UAE using gelatin sponge particles. The treatment was successful and this patient had a normal pregnancy [29]. We recommend that obstetricians should be aware of the possible existence of severe side effects so that careful measures can be taken.

Among the limitations of this research is the relatively small-sample (61 participants in total) randomized controlled trials as it was limited to Shanghai Sixth People’s Hospital Affiliated to Shanghai Jiaotong University. Some follow-up information extracted from the charts was incomplete, particularly regarding blood routine examination, liver and kidney function test, time of CSP residual mass disappearance, time of serum β-hCG resolution, and reproductive outcome. In order to establish its evidence-based role in the time interval between UAE and D&C, clinical trials should be carried out, on a wider range of patients with CSP, to endorse practical recommendations.

## Conclusions

In summary, both groups were successful in terminating CSP and each had clinical advantages. For patients with CSP, UAE within 12 h followed by D&C appears to have more advantages in hospitalization and recovery time, while the interval at 12–72 h may have a lower risk of inflammation and a faster decrease rate of serum β-hCG level within 24 h after D&C surgery. The treatment of CSP should be individualized and some conditions must be considered based on risk factors since each patient presents differently. After multidisciplinary clinical assessment, the treating obstetricians should counsel the patient with CSP regarding the different regimens of treatments provided by the hospital, their advantages, and disadvantages [10, 30].

## Data Availability

Data supporting the findings of this study are available from the corresponding author (Zhuowei Xue) upon reasonable request.

## Abbreviations

BMI: Body mass index
CNY: China yuan
CRP: C-reactive protein
CS: Cesarean section
CSP: Cesarean scar pregnancy
D&C: Dilatation and curettage
MRI: Magnetic resonance imaging
SD: Standard deviation
UAE: Uterine artery embolization
β-hCG: Beta human chorionic gonadotropin.
